# Pig Passage Counting Based on Improved YOLO and HMTC Strategy

**DOI:** 10.3390/ani16131951

**Published:** 2026-06-24

**Authors:** Lu Yang, Saisai Wu, Shuqing Han, Xin Chai, Yali Wang, Hongyu Zhang, Guodong Cheng

**Affiliations:** 1 Agricultural Information Institute, Chinese Academy of Agricultural Sciences, Beijing 100081, China; yanglu01@caas.cn (L.Y.); hanshuqing@caas.cn (S.H.); chaixin@caas.cn (X.C.); wangyali01@caas.cn (Y.W.); zhanghongyu@caas.cn (H.Z.); 2 Key Laboratory of Agricultural Blockchain Application, Ministry of Agriculture and Rural Affairs, Beijing 100081, China; 3 Guangxi Academy of Agricultural Sciences, Nanning 530007, China; wusaisai0324@163.com

**Keywords:** pig counting, object detection, YOLOv11, livestock management

## Abstract

Accurate pig counting during herd transfers is fundamental to effective livestock management in large-scale swine production, yet existing methods struggle with bidirectional passages, boundary oscillations, and occlusion in real corridor environments. This study proposes an integrated system combining an improved YOLO-based detection model with a Hysteresis-based Multi-frame Temporal Confirmation Counting Strategy (HMTC). The YOLO11s baseline was enhanced using lightweight RepViT blocks, dynamic upsampling (DySample), and shape-aware bounding box regression (Shape-IoU). The resulting model achieves a mAP_50_ of 0.982 with a compact architecture of 8.28M parameters, representing a 12.3% reduction relative to the baseline while improving detection accuracy. To address bidirectional counting challenges, the HMTC strategy utilizes hysteresis-based region classification, temporal confirmation, and trajectory verification to suppress boundary jitter and ensure directional correctness. Evaluated on nine videos from a single transfer corridor, the proposed system achieves an overall counting accuracy of 99.21% on this test set and runs in real time on an embedded edge device at over 30 FPS without loss of counting accuracy. Together, the improved detection model and HMTC counting strategy provide a cohesive approach to pig passage counting, validated here under a single transfer-corridor condition; these results offer a promising basis for automated animal inventory management, pending further validation across more diverse farm environments.

## Simple Summary

Pigs are active and social animals that rarely stand still, often pushing, huddling, and moving unpredictably, which makes accurate counting a persistent challenge in daily farm management. These natural behaviors, combined with motion blur, often lead to errors in both manual and automated counting. To address this, we developed an automated camera-based system that combines an improved detection model with a specialized counting strategy specifically designed for real farm transfer corridors. The system accurately identifies individual pigs in tightly packed groups and reliably tracks their movement directions. Tested on nine videos recorded from a single transfer corridor, the system counted pigs’ accurately and reliably registered backward movements, indicating its potential as a non-invasive aid for animal inventory management. Further validation across diverse farms, breeds, and corridor layouts is required for broader application.

## 1. Introduction

Pig farming is a cornerstone of global meat production, with swine representing one of the most significant livestock species worldwide [1]. In China, pork production reached a record 59.38 million tonnes in 2025, accounting for approximately 59% of the nation’s total meat output of 100.72 million tonnes [2]. As farm operations scale up to meet growing demand, effective herd management has become increasingly critical for maintaining production efficiency, disease surveillance, and animal welfare standards [3]. Among these, accurate pig counting during herd transfers represents a fundamental yet operationally demanding task in large-scale swine production [4]. Manual counting is not only time-consuming and error-prone, but also induces stress responses in animals due to repeated human interaction, potentially compromising their health and productivity [5]. As farm scales continue to expand, these limitations have catalyzed the transition toward computer vision-based automated systems, which provide non-invasive, consistent, and real-time monitoring during routine husbandry operations [6,7]. This shift reflects a broader trend in which the Augmented Intelligence of Things (AIoT) and edge computing are increasingly applied to real-time sensing, trajectory analysis, and automated decision-making across domains [8].

Existing vision-based approaches for pig counting can be broadly categorized into two paradigms. The first treats counting as a static image-based detection problem, focusing on identifying and localizing individual animals within single frames to estimate population density in enclosed pens [9]. Luo et al. proposed a lightweight YOLOv8-seg model for automatic pig counting by incorporating the Ghost module and a spatial group-enhanced attention mechanism, which achieved an average counting precision of 95.7% [10]. Similarly, Li et al. improved the YOLOv8 with DCNv4 and BiFPN to handle varying pig postures [11], while He et al. introduced the PDC-YOLO with SPD-Conv for lightweight and AFPN for efficiently feature fusing [12], achieving high counting accuracies of 96.8% and 95.1%, respectively. With the development of object detection models, especially the YOLO series, the method of counting by detection has substantially advanced detection performance under diverse lighting conditions and moderate occlusion, achieving highly accuracy of generally exceeding 90% in controlled pen environments [13,14,15]. Nevertheless, static methods are inherently limited by their lack of temporal context. They cannot distinguish unique individuals from re-entries, and remain susceptible to frequent movement or overlapping behaviors, rendering them unsuitable for continuous passage monitoring.

Complementary to static detection-based methods, some research adopts video-based counting through multi-object tracking, which maintains individual identities and records crossing events by associating detections across consecutive frames. For instance, Kim et al. deployed a lightweight detection-and-tracking pipeline on an NVIDIA Jetson Nano embedded board, achieving a counting accuracy of 99.44% for pigs passing through a designated counting zone [16]. Shao et al. combined an improved YOLOv7 with DeepSORT, incorporating coordinate attention and partial convolution to enhance robustness under oblique viewing angles, attaining an average counting accuracy of 96.58% across multiple corridor scenarios [17]. Similarly, Shen et al. integrated ELA attention and GSConv into YOLOv8n, and augmented Deep SORT with DenseNet-based feature extraction and CIoU matching, yielding a counting accuracy of 92.1% with notably reduced identity switches [18]. Collectively, these works demonstrate that coupling improved detectors with multi-object trackers provides a viable foundation for automated pig counting in structured corridor environments.

Notwithstanding the progress in both static and tracking-based approaches, robust pig counting in real transfer corridor scenarios remains a persistent challenge. Reliable detection forms the foundation of any robust counting system, directly influencing both counting precision and system deployability [19]. While the YOLO series is widely used in livestock monitoring applications for its balance of accuracy, speed and usability [20,21,22], achieving robust detection in pig transfer corridor scenarios remains difficult [23]. Challenges such as low animal-background contrast, motion blur, and dense occlusion place stringent demands on detection models. Beyond detection, pig transfers in commercial production often involve highly irregular movement patterns, including prolonged boundary lingering and frequent directional reversals [24]. Existing counting strategies face characteristic limitations in dense transfer scenarios. Line-crossing counting is sensitive to boundary jitter and tends to double-count oscillating animals; region-based counting reduces jitter but cannot reliably distinguish genuine reverse crossings from momentary back-and-forth movements; and tracking-based solutions commonly lack a dedicated mechanism to validate crossing direction, so reverse movements of previously counted individuals or transient identity switches are mis-registered, resulting in unreliable bidirectional counting that limits practical applicability in real farm transfer scenarios [25].

To jointly address these challenges, this study proposes an improved YOLO-based detection model by incorporating three modifications into the YOLO11s baseline, which comprise a C3k2_RepViT module that replaces standard bottleneck units with lightweight RepViT blocks to enhance feature extraction efficiency [26], a DySample upsampling module that improves multi-scale feature representation through content-aware dynamic sampling [27], and Shape-IoU as the bounding box regression loss to improve localization precision under varying pig postures and occlusion patterns [28]. Second, a Hysteresis-based Multi-frame Temporal Confirmation counting strategy is proposed to address the bidirectional counting challenge inherent to real farm transfer scenarios. HMTC integrates hysteresis-based region classification to suppress boundary jitter, multi-frame temporal confirmation to filter transient tracking noise, and trajectory-based verification to ensure reliable directional registration. Together, these two components form a cohesive system for automated pig passage monitoring, which we validate in this study as a preliminary proof of concept under a single transfer-corridor condition.

## 2. Materials and Methods

### 2.1. Pig Data Collection and Curation

#### 2.1.1. Data Collection

**Figure 1:**
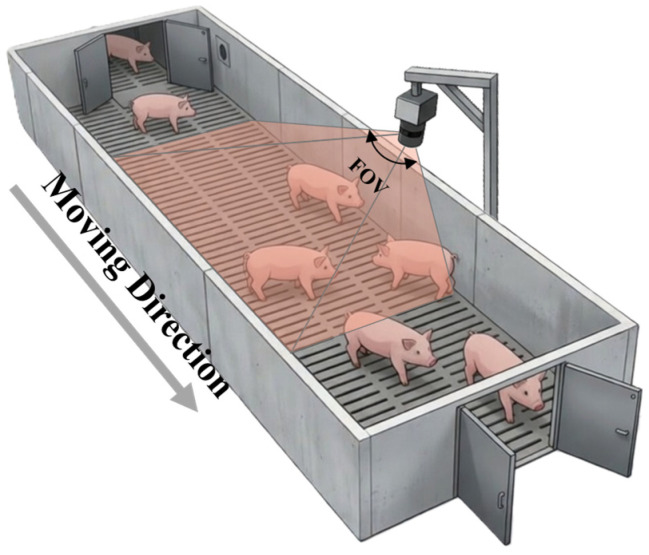
Schematic diagram of the data collection in the pig transfer corridor.

The raw pig video data used in this study were collected from the Daweijia Pig Farm, located in Pinggu District, Beijing, China. As illustrated in Figure 1, a fixed-position camera was mounted overhead within the transfer corridor to capture a top-down view of the natural locomotion and passage behavior of the pigs. The corridor was approximately 1.5 m wide, and the experimental subjects were Landrace weaned piglets at approximately 28 days of age, corresponding to the weaning and transfer stage.

A series of video sequences were recorded under natural lighting conditions at a resolution of 3840 × 2160 pixels and a frame rate of 60 frames per second (fps). The collected footage encompasses a wide range of scenarios, ensuring the diversity and representativeness of the dataset for subsequent model training and evaluation.

**Figure 2:**
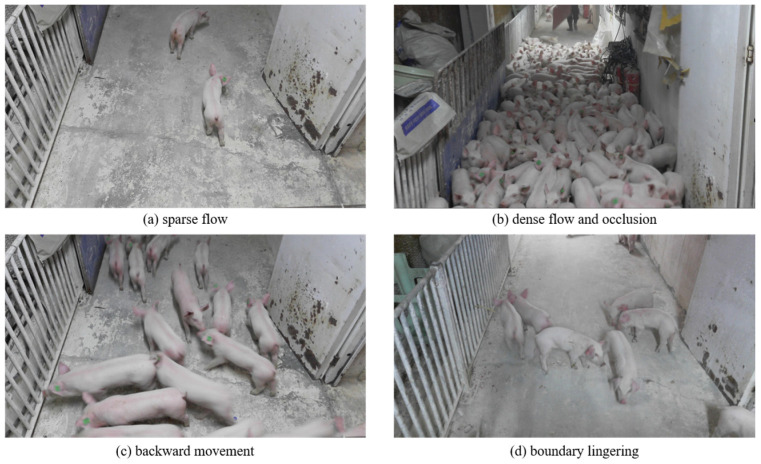
Representative raw frames from the dataset illustrating the principal challenges of corridor-based pig counting. (**a**) sparse flow; (**b**) dense flow with severe occlusion; (**c**) backward movement against the main flow; (**d**) boundary lingering.

Representative raw frames illustrating the principal challenges of the dataset are shown in Figure 2. The corridor scenario varies greatly in flow density, ranging from sparse passage of a few individuals (Figure 2a) to dense, tightly packed flow in which animals heavily occlude one another (Figure 2b), making individual detection and identity association difficult. In addition, pigs frequently move backward against the main flow (Figure 2c) and linger near the corridor boundaries without committing to a crossing (Figure 2d); both behaviors are common sources of miscounting for conventional boundary-based methods. These conditions make accurate counting in the transfer corridor a non-trivial task and motivate the detection and counting strategies described in the following sections.

#### 2.1.2. Frame Extraction and Redundancy Removal

Individual frames were extracted from the raw video sequences to construct the initial pig detection dataset. However, given the high frame rate of 60 fps adopted during recording, consecutive frames exhibit an extremely high degree of visual similarity, resulting in substantial inter-frame redundancy. Therefore, a similarity-based filtering pipeline was developed to systematically eliminate redundant frames.

Inter-frame similarity was evaluated using the Structural Similarity Index Measure (SSIM) [29], which provides a perceptually meaningful measure of luminance, contrast, and structural consistency between adjacent frames. To balance computational efficiency and filtering accuracy, a sliding window strategy was adopted, wherein each candidate frame was compared exclusively against the most recently retained frames within a fixed-size window. When the SSIM score between a candidate frame and any frame within the window exceeded the similarity threshold of 0.95, the candidate was classified as a near-duplicate and discarded.

Following this redundancy removal pipeline, the dataset size was substantially reduced while preserving the representative diversity of pig movement scenarios, providing a clean and balanced foundation for subsequent annotation and model training.

#### 2.1.3. Image Annotation and Dataset Partition

For object detection, all individual pigs visible in each frame were manually annotated with bounding boxes using X-AnyLabeling [30], a semi-automatic annotation tool that integrates AI-assisted labeling to accelerate the annotation workflow. Annotations were exported in YOLO format. All annotations were subsequently reviewed and verified to minimize labeling errors, ensuring the accuracy and consistency of the detection ground truth.

For passage counting, a virtual detection line was defined at one-third of the image height from the top of the frame, corresponding to a fixed transverse position within the transfer corridor. This line serves as the reference boundary for determining passage events: a pig is recorded as having passed through the corridor when its trajectory crosses the virtual line over consecutive frames. The temporal crossing records were aggregated to generate cumulative passage counts, forming the ground truth for the counting task.

The finalized dataset comprises nine video sequences with durations ranging from 12 to 99 s, totaling 5652 annotated frames. As summarized in Table 1, the videos cover a wide range of passage densities. The number of pigs visible per frame ranges from 1 to 57, and the total number of crossing events per video ranges from 9 to 76 (forward 7–64, backward 0–22). This variability in both density and directional flow ensures that the detection and counting components of the proposed system are evaluated under sufficiently challenging and representative conditions.

To train the YOLO-pig detection model, the annotated dataset was partitioned at the video level rather than by random frames, so as to prevent leakage arising from the high visual similarity between adjacent frames. Videos 1–7 (4814 frames) were used for training and videos 8–9 (838 frames) for validation, ensuring that frames from the same video never appear in both subsets.

### 2.2. Construction of Pig Detection Model Based on Improved YOLOv11

#### 2.2.1. Improved C3k2_RepViT for Feature Extraction

The backbone and neck of YOLO11 rely on convolutional C3k2 modules for hierarchical feature extraction. While effective, these convolution-dominant structures are inherently limited in modeling long-range spatial dependencies and adaptive feature interactions. Direct integration of Vision Transformer (ViT) components can enhance representation capacity, yet such approaches introduce substantial computational overhead that is incompatible with real-time edge deployment in livestock monitoring systems.

**Figure 3:**
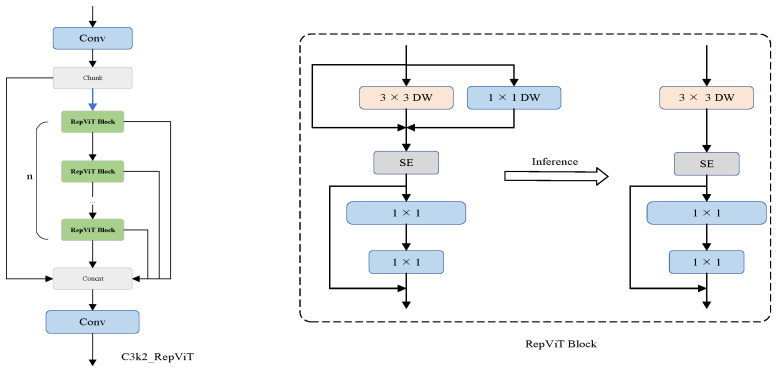
Structure of C3k2_RepViT.

As shown in Figure 3, we replace the Bottleneck units within the C3k2 module with RepViT blocks [26], forming an enhanced structure termed C3k2_RepViT. RepViT is a lightweight MetaFormer-style architecture that decouples spatial and channel feature mixing. Specifically, spatial interaction is conducted via 3 × 3 depthwise convolution, while channel mixing is implemented using 1 × 1 pointwise convolution with moderate expansion. This decoupled design improves feature modeling flexibility without increasing computational burden.

A critical property of RepViT is its structural re-parameterization mechanism. During training, multi-branch convolutional paths improve representation diversity and gradient flow. At inference, these branches are analytically fused into a single convolutional layer, ensuring that enhanced training-time expressiveness does not incur additional inference latency.

By integrating RepViT into YOLO11, the proposed C3k2_RepViT module strengthens contextual modeling and multi-scale representation while preserving real-time performance. This design is particularly advantageous for pig detection in dense and occluded farm environments, where robust feature discrimination must be achieved under strict computational constraints.

#### 2.2.2. Ultra Lightweight Upsample

**Figure 4:**
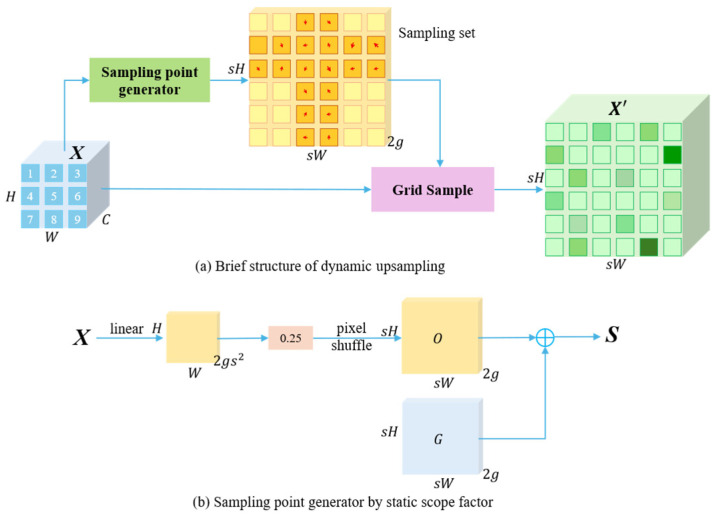
Brief structure of DySample and its implementation of scope factor. (**a**) brief structure of upsampling; (**b**) sampling point generator by static scope factor.

To enhance the feature upsampling quality in the neck network, we replace the traditional nearest neighbor (NN) upsampling layers in YOLO with DySample, a lightweight dynamic upsampling module [27]. As shown in Figure 4, unlike conventional interpolation methods that follow fixed rules, DySample reformulates upsampling from the perspective of content-aware point sampling.

Specifically, given an input feature map $X\in R^{C\times H\times W}$ and upsampling factor s, DySample first generates content-aware sampling offsets $O\in R^{2{gs}^{2}\times H\times W}$ through a linear projection:

$$O=\mathrm{Linear}\left(X\right)$$

where the linear layer maps C input channels to $2{gs}^{2}$ output channels (2 for x/y coordinates, ${gs}^{2}$). The predicted offsets are then rearranged via pixel shuffle to produce $O^{\prime }\in R^{2\times sH\times sW}$. The final sampling grid is obtained by adding the offsets to a predefined base grid G:

$$S=G+O^{\prime }$$

Finally, the upsampled feature $X^{\prime }\in R^{C\times sH\times sW}$ is obtained via PyTorch’s (2.7.1) built-in grid_sample:

$$X^{\prime }=\mathrm{grid}\_\mathrm{sample}\left(X,S\right)$$

DySample provides two strategies to regulate the offset magnitude. The **dynamic-scope version** introduces an additional linear branch to predict a content-adaptive modulation factor:

$$O=0.5\cdot \sigma \left({\mathrm{linear}}_{1}\left(X\right)\right)\cdot {\mathrm{linear}}_{2}\left(X\right)$$

where the sigmoid activation constrains the modulation range. This design allows spatially adaptive control of sampling offsets but increases computational complexity. In this work, we adopt the **static-scope version**, where the predicted offsets are scaled by a fixed coefficient:

$$O=0.25\cdot \mathrm{Linear}\left(X\right)$$

The constant scaling factor restricts the offset range, preventing excessive overlap between neighboring sampling regions while maintaining lightweight computation and stable optimization, which is more suitable for real-time detection scenarios.

#### 2.2.3. Shape-IoU for Accurate Bounding Box Regression

**Figure 5:**
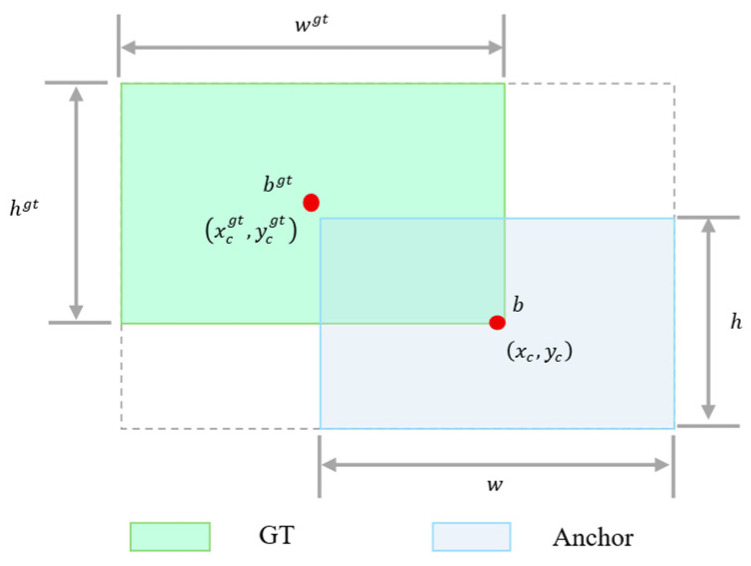
Geometric illustration of the Shape-IoU calculation.

To further enhance bounding box regression accuracy, especially for small and shape-sensitive targets such as pigs, we introduce Shape-IoU [28], a shape- and scale-aware localization metric. As shown in Figure 5, unlike conventional IoU-based losses that primarily focus on geometric relationships (e.g., overlap area, center distance, or aspect ratio difference), Shape-IoU explicitly models the intrinsic shape and scale properties of the ground-truth bounding box itself. The calculation formulas were presented as follows:

$$IoU=\frac{\left|B\cap B^{gt}\right|}{\left|B\cup B^{gt}\right|}$$

To incorporate shape sensitivity, Shape-IoU introduces direction-aware weighting coefficients based on the ground-truth box dimensions, as ww and hh,

$$ww=\frac{2\times (w^{gt}{)}^{scale}}{\left(w^{gt}{)}^{scale}+(h^{gt}{)}^{scale}\right.}$$

$$hh=\frac{2\times (h^{gt}{)}^{scale}}{\left(w^{gt}{)}^{scale}+(h^{gt}{)}^{scale}\right.}$$

and the center-distance penalty $distance_{shape}$ is reweighted as:

$$distance_{shape}=hh\frac{\left(x_{c}-x_{c}^{gt}{)}^{2}\right.}{c^{2}}+ww\frac{\left(y_{c}-y_{c}^{gt}{)}^{2}\right.}{c^{2}}$$

where c is the diagonal length of the smallest enclosing box.

Shape-IoU incorporates a shape consistency penalty term $\Omega_{shape}$:

$$\Omega_{shape}=\sum_{t\in \{w,h\}}{\left(1-e^{-\omega_{t}}\right)}^{\theta }$$

$$\omega_{w}=hh\frac{\left|w-w^{gt}\right|}{\max\left(w,w^{gt}\right)}$$

$$\omega_{h}=ww\frac{\left|h-h^{gt}\right|}{max(h,h^{gt})}$$

and the final Shape-IoU regression loss is defined as:

$$L_{Shape\text{-}IoU}=1-IoU+distance_{shape}+0.5\cdot \Omega_{shape}$$

By explicitly incorporating shape-aware weighting and consistency constraints, the loss function guides the model to produce bounding boxes that not only overlap well with the ground truth but also preserve the correct shape and scale properties. This is particularly beneficial for pig detection tasks, where animals frequently exhibit diverse postures, partial occlusions, and varying body orientations that challenge conventional localization methods.

**Figure 6:**
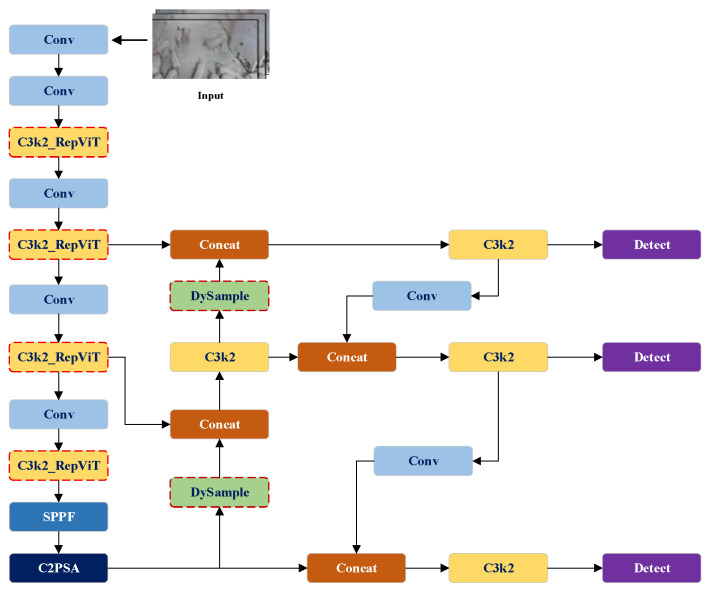
Overall architecture of the proposed YOLO-pig detection model.

Together, the overall architecture of the proposed YOLO-pig model is illustrated in Figure 6. Building upon the standard YOLO11s architecture, the C3k2 modules in the backbone are replaced with C3k2_RepViT, and the conventional upsample operations in the neck are substituted with DySample modules. All remaining components retain the original YOLO11 structure. Additionally, Shape-IoU is introduced as the bounding box regression loss during training to improve localization precision.

### 2.3. HMTC: Hysteresis-Based Multi-Frame Temporal Confirmation Counting Strategy

While the improved YOLO-pig model provides accurate per-frame pig detection, reliable passage counting also requires associating detections across frames and handling real-world challenges such as boundary jitter, frequent occlusions, and irregular movements. To this end, the per-frame detections are first associated into trajectories using the ByteTrack tracker, which assigns a persistent identity to each pig. Operating on these trajectories, we propose the HMTC strategy, which integrates three complementary components to ensure robust bidirectional counting: hysteresis-based region classification to suppress boundary jitter, multi-frame temporal confirmation to reject single-frame noise, and trajectory-based verification to distinguish legitimate reverse crossings from transient tracking noise. Each component is detailed in the following subsections.

#### 2.3.1. Hysteresis-Based Region Classification

To mitigate the “boundary jitter” caused by detection noise or pig head movements near the classification lines, we implement a hysteresis-based region classification method inspired by the Schmitt trigger. As shown in Figure 5, the monitoring area was divided into three zones: Up, Mid, and Down, separated by two baseline thresholds $T_{up}$ and $T_{down}$. Unlike static thresholds, the specific region assignment $R_{t}$ at time t depends not only on the current vertical coordinate $y_{t}$ but also on the target’s previous stable region $R_{prev}$. The state transition logic for the $R_{t}$ is defined as follows:

$$R_{t}=\left\{\begin{array}{ll} Up, & \mathrm{if}\;R_{prev}=Mid\;\mathrm{and}\;y_{t}<(T_{up}-\Delta )\\ Down, & \mathrm{if}\;R_{prev}=Mid\;\mathrm{and}\;y_{t}\geq T_{down}\\ Mid, & \mathrm{if}\;R_{prev}=Up\;\mathrm{and}\;y_{t}\geq T_{up}\\ Mid, & \mathrm{if}\;R_{prev}=Down\;\mathrm{and}\;y_{t}<(T_{down}-\Delta )\\ R_{t-1}, & \mathrm{otherwise} \end{array}\right.$$

where $T_{up}$ and $T_{down}$ represent the baseline boundaries of the passage, and Δ denotes the hysteresis band width.

**Figure 7:**
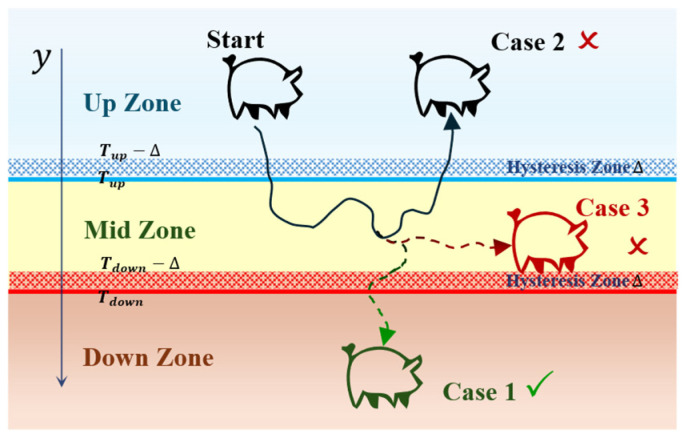
Illustration of the three-zone partition and hysteresis mechanism in HMTC.

As shown in Figure 7, which illustrates the hysteresis mechanism through three typical scenarios. In Case 1 (standard passage), when a pig moves from UP and crosses $T_{up}$, it enters MID and proceeds toward DOWN. In Case 2 (boundary oscillation), minor retreats above $T_{up}$ do not trigger state reversion as long as the coordinate remains above $T_{up}-\Delta$; the hysteresis band (blue shaded region) absorbs these fluctuations, preventing false transitions. In Case 3 (prolonged dwelling), the pig lingers within the MID zone $\left[T_{up}-\Delta ,T_{down}\right]$ for an extended period, with the system maintaining the “MID” state to enable subsequent temporal confirmation of the exit direction. This asymmetric threshold design provides spatial stability, ensuring that only confirmed region transitions propagate to the counting stage.

#### 2.3.2. Multi-Frame Temporal Confirmation

**Figure 8:**
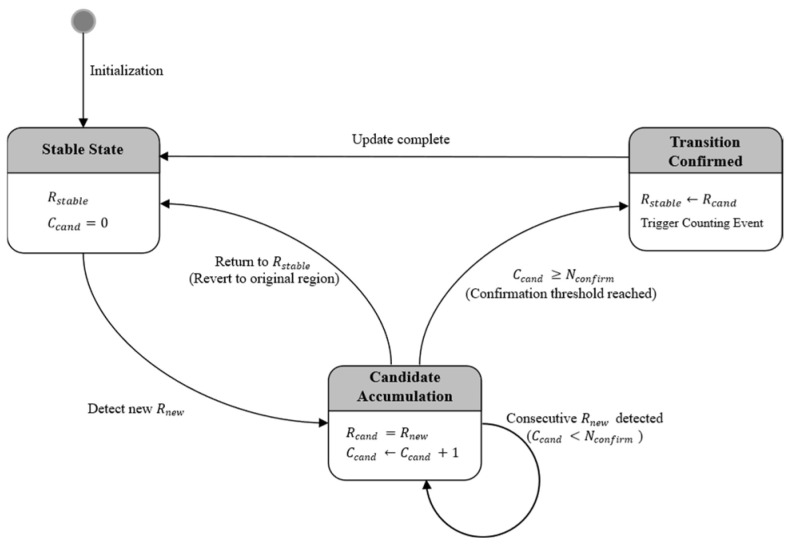
Finite State Machine for pig counting region transition.

Even with hysteresis, single-frame region transitions remain susceptible to transient occlusions. HMTC introduces a temporal confirmation layer to validate the stability of region changes. As shown in Figure 8, the system utilizes a Finite State Machine (FSM) with a candidate-confirmation mechanism:

**Stable State:** The pig is assigned to a confirmed region $R_{stable}$. The system remains in this state as long as the current classification $R_{t}$ matches $R_{stable}$.

**Candidate Accumulation:** When $R_{t}\neq R_{stable}$, a candidate region $R_{cand}$ is initialized. A confirmation counter $C_{cand}$ increments for each consecutive frame where the detected region remains $R_{cand}$.

**Transition Confirmed:** A transition is only finalized if $C_{cand}$ reaches the threshold $N_{confirm}$ (e.g., 2 frames).

The counting event is triggered only upon a confirmed transition between stable regions, ensuring that only intentional movements are recorded.

State transition diagram of the multi-frame confirmation mechanism principle in circuit design:

$$R_{stable}^{t}=\left\{\begin{array}{cc} R_{cand}^{t} & \mathrm{if}\;C_{cand}^{t}\geq N_{confirm}\\ R_{stable}^{t-1} & \mathrm{otherwise} \end{array}\right.$$

We introduce a hysteresis mechanism to eliminate boundary jitter. Unlike conventional hard-threshold classification that uses fixed values for region transitions, our method employs asymmetric thresholds depending on the previous stable region.

#### 2.3.3. Direction Inference and Anomaly Handling

To further refine accuracy, HMTC integrates trajectory-based verification and an anomaly self-recovery mechanism.

**A. Trajectory-Based Verification:** The system validates movement using a position history buffer P. For any confirmed transition, the system calculates the displacement trend $v_{overall}=\left(y_{last}-y_{first}\right)/\left(t_{last}-t_{first}\right)$. A counting event is valid only if the displacement sign aligns with the transition direction (e.g., $v_{overall}>0$ for Up →Mid →Down). This filters out “false transitions” caused by pigs standing near the boundary.

**B. Anomaly Handling and Self-Recovery:** The strategy monitors the instantaneous velocity $v_{inst}$ to maintain tracking integrity. If $\left|v_{inst}\right|$ exceeds a physical limit $V_{max}$ or exhibits radical direction reversals, an anomaly is flagged. Upon detecting $N_{max}$ consecutive anomalies (e.g., 5 frames), the system assumes a tracking failure, triggers a state reset, and clears the trajectory history to prevent erroneous cumulative counts.

#### 2.3.4. Implementation Details and Parameter Settings

Before the HMTC strategy is applied, the per-frame detections produced by YOLO-pig are associated across consecutive frames into trajectories using the ByteTrack tracker, which performs robust motion-based association without relying on appearance features and assigns a persistent identity to each pig. The resulting trajectories are then processed by the three HMTC components described above. The complete set of HMTC parameters, together with the basis for selecting each value, is summarized in Table 2.

All parameters were set empirically based on the corridor geometry and the processing frame rate, and were kept fixed across all videos and experiments. Here, w and h denote the frame width and height, respectively; thresholds defined relative to the frame size make the configuration independent of the input resolution.

### 2.4. Edge Deployment Setup

To evaluate practical deployability on farm-side hardware, the complete detection-and-counting pipeline was deployed on an NVIDIA Jetson Orin NX (16 GB; JetPack 6.2.1, TensorRT 10.3), operated in the MAXN_SUPER power mode with locked clocks for reproducible measurements. The trained detector was converted from PyTorch to ONNX and then compiled into a TensorRT FP16 engine on the device, while object association and counting reused the same ByteTrack tracker and HMTC strategy as in the workstation pipeline; the detection input was kept at 640 × 640, identical to training.

The pipeline was profiled per frame across six stages—decode, preprocess, inference, postprocess, track association and result transfer, and HMTC—with software video decoding. Profiling was performed at the 1080p deployment resolution and at native 4K under identical settings, using the sparsest and densest sequences in the dataset to span the range of scene densities. Each configuration was averaged over three full runs after a 30-frame warm-up. Memory usage was measured as the increment over idle (jtop), and edge counting outputs were verified against the workstation results on the same sequences.

### 2.5. Evaluation Metrics

**Detection Performance:** The detection performance of YOLO-pig is evaluated using precision (P), recall (R), and mean average precision at IoU thresholds of 0.50 and 0.50:0.95 (mAP_50_ and mAP_50-95_). mAP_50-95_ serves as the primary indicator of bounding box localization quality, as it averages performance across stricter IoU thresholds and is more sensitive to precise spatial alignment.

**Counting Performance:** Counting accuracy is assessed using four metrics across forward and backward directions separately. For each video, the predicted count (Pred) is compared with the ground-truth count (GT) to obtain true positives TP = min(GT, Pred), false positives FP = max(0, Pred − GT), and false negatives FN = max(0, GT − Pred), where FP and FN correspond to over- and under-counting, respectively. The absolute error is AE = |Pred − GT| = FP + FN. The overall counting accuracy is defined as a count-weighted measure aggregated over all videos:

ACC=1−(∑AE)/(∑GT)

which is well-defined regardless of any individual zero-count direction and removes the need for the small constant used previously. For directions with GT = 0, no ratio is computed and the false-positive count is reported directly. TP, FP, FN, AE, and ACC are reported separately for the forward and backward directions as well as in aggregate.

## 3. Results

### 3.1. Performance of YOLO Pig Detection Model

All experiments were conducted on a workstation equipped with an AMD Ryzen 5 7500F CPU, 32 GB RAM, and an NVIDIA GeForce RTX 4070 GPU. The deep learning framework used was PyTorch 2.7.1 with CUDA 12.6. All models were trained for up to 500 epochs with a batch size of 16 and an input resolution of 640 × 640 pixels. Early stopping was applied with a patience of 50 epochs, and mosaic augmentation was disabled during the final 30 epochs to stabilize convergence.

To determine an appropriate baseline for subsequent improvements, four scale variants of YOLO11—YOLO11n, YOLO11s, YOLO11m, and YOLO11l—were evaluated on the pig detection dataset under identical training configurations. Detailed per-variant metrics are provided in Appendix A Table A1. Considering the trade-off between detection accuracy and inference efficiency required for real-time livestock monitoring, YOLO11s was selected as the baseline model for all subsequent experiments.

#### 3.1.1. Comparison of IoU Loss Functions

To validate the effectiveness of Shape-IoU, we compared four IoU-based regression losses under identical training configurations. As shown in Table 3, Shape-IoU achieves the highest mAP_50-95_ of 0.813, outperforming CIoU (0.791), GIoU (0.790), and DIoU (0.783) by a notable margin. Although its mAP_50_ (0.975) is marginally lower than CIoU (0.978), the improvement in mAP_50-95_ indicates that Shape-IoU produces geometrically more precise bounding boxes by explicitly incorporating ground-truth shape and scale information, which would directly benefit the downstream trajectory-based counting in HMTC. Shape-IoU was therefore adopted in all subsequent experiments.

#### 3.1.2. Detection with Improved YOLO

We conducted ablation experiments to evaluate the individual and combined contributions of C3k2_RepViT and DySample, using YOLO11s with Shape-IoU as the regression loss baseline. The results are summarized in Table 4.

As shown in Table 3, incorporating C3k2_RepViT alone reduces the parameter count from 9.41M to 8.25M (−12.3%) and GFLOPs from 21.3 to 19.3 (−9.4%), with only negligible changes in detection accuracy, demonstrating that replacing the standard C3k2 bottleneck with RepViT blocks effectively reduces model complexity while preserving feature extraction capability. Adding DySample alone yields consistent accuracy improvements, with mAP_50_ increasing to 0.977 and mAP_50-95_ to 0.825, confirming that content-aware dynamic upsampling provides richer multi-scale feature representations compared to the default nearest-neighbor interpolation.

The full model combining both modules achieves the best mAP_50_ of 0.982 with a relatively compact architecture (8.28M parameters, 19.4 GFLOPs), demonstrating a favorable accuracy–efficiency trade-off compared to the baseline. Although mAP_50-95_ (0.820) is marginally lower than DySample-only (0.825), this minor variation is offset by the simultaneous gains in mAP_50_ and the substantial reduction in model complexity. The proposed YOLO-pig model therefore achieves improved detection performance with a leaner architecture, making it well-suited for real-time deployment in livestock monitoring scenarios.

**Figure 9:**
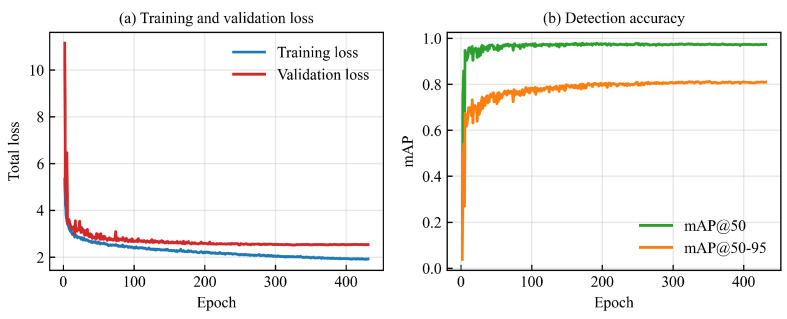
Training curves of the proposed YOLO-pig model: (**a**) training and validation total loss and (**b**) mAP@50 and mAP@50-95 over training epochs.

The training process of the proposed YOLO-pig model is shown in Figure 9. Both the training and validation losses decrease rapidly in the early epochs and then converge to a stable plateau, with the validation loss closely tracking the training loss and exhibiting no upward trend, while mAP@50 and mAP@50-95 rise and saturate in the later epochs. These curves confirm that the model converges stably without overfitting.

### 3.2. Comparative Results of Pig Passage Counting Methods

Three counting methods—Line (YOLO), Region (YOLO), and HMTC—were compared across the nine test videos using the metrics defined in Section 2.4. To isolate the effect of the counting strategy itself, all three methods share the same backbone—the YOLO-pig detector combined with the ByteTrack tracker—and differ only in how the resulting trajectories are converted into directional counts. The line- and region-based methods correspond to the official object-counting solutions provided in the Ultralytics framework, whereas HMTC replaces only the counting logic. The test videos cover a range of real-world passage scenarios, including sparse and dense pig flows, varying forward-to-backward ratios, and cases with no backward movement, providing a comprehensive basis for evaluating counting robustness across diverse conditions.

**Figure 10:**
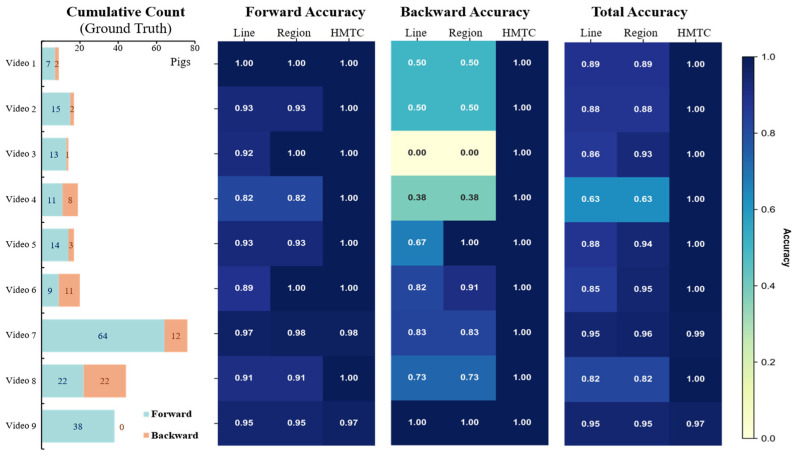
Pig passage counting performance across nine test videos.

As shown in Figure 10, HMTC achieves perfect forward accuracy (1.00) across seven of the nine videos, with Video 7 and Video 9 recording 0.98 and 0.97, respectively, and attains a perfect backward accuracy of 1.00 across all nine videos. In contrast, Line (YOLO) and Region (YOLO) exhibit notable deficiencies in backward counting, with accuracies as low as 0.00 (Video 3) and 0.38 (Video 4), reflecting their susceptibility to boundary jitter and transient occlusions during reverse passages.

The aggregated results in Table 5 further confirm the superiority of HMTC. The line-based and region-based methods incur total absolute errors of 31 and 26 crossings, corresponding to overall counting accuracies of 87.80% and 89.76%, respectively. Their errors are heavily concentrated in the backward direction, where they accumulate backward absolute errors of 19 and 17—far exceeding their forward errors of 12 and 9. This stems from an ID-based design constraint: once an individual has been counted as crossing the detection line or region in one direction, any subsequent crossing in the opposite direction by the same track ID is not registered, leading to systematic under-counting of reverse movements regardless of the backward traffic volume; the effect is most pronounced in videos with heavy reverse flow such as videos 4 and 8. In contrast, HMTC reduces the total absolute error to only 2 crossings, yielding an overall accuracy of 99.21% on the current test set (ΣFP = 0, ΣFN = 2). Notably, it produces no backward miscounts across all nine videos (backward absolute error of 0), and its only two errors are single under-counts (false negatives) occurring in the two densest sequences (videos 7 and 9). These results demonstrate that the hysteresis-based region classification, multi-frame temporal confirmation, and trajectory-based verification are jointly essential for reliable bidirectional counting, particularly for the backward crossings that the baseline methods fail to capture.

Moreover, a closer inspection of the YOLO object counting solution source code reveals that once a tracked individual crosses the detection line or region in either direction, any subsequent crossing in the opposite direction by the same track ID is not registered, leading to systematic undercounting of reverse movements.

### 3.3. Edge Deployment Evaluation

To assess practical deployability on farm-side hardware, the improved detector was exported to ONNX and compiled to a TensorRT FP16 engine on an NVIDIA Jetson Orin NX (16 GB, JetPack 6.2.1, TensorRT 10.3, MAXN_SUPER with jetson_clocks). The complete pipeline (decoding, preprocessing, detection, NMS, ByteTrack association, and HMTC) was benchmarked on real corridor video; each configuration was averaged over three runs.

To characterize performance under the most demanding conditions, we report results on the densest sequence, video7 (64 forward and 12 backward crossings), so that throughput on sparser sequences is correspondingly higher. As summarized in Table 6, on this densest sequence the system processes 1080p video end-to-end at 30.4 FPS (32.87 ms/frame) with an incremental memory footprint below 1 GB, while detection inference alone accounts for only 7.5 ms/frame. Processing at native 4K is markedly slower (12.6 FPS), as the additional cost lies almost entirely in resolution-dependent frame decoding and preprocessing rather than in network inference (see the per-stage breakdown in Appendix A Table A2), which is essentially unaffected by source resolution.

Frames are sampled at a 2:1 ratio from the 60-fps stream, giving an effective processing rate of 30 fps. The 1080p throughput therefore meets the required rate even on the densest sequence; across the densest (video7) and sparsest (video1) sequences, the end-to-end rate ranged from 30.4 to 32.8 FPS, bounding the throughput for all intermediate densities. On video7, the forward/backward counts on the edge device were identical to the workstation results (63/12) and unchanged between 1080p and 4K, indicating that FP16 conversion and edge execution do not alter counting behavior. These results demonstrate real-time on-device operation across the observed range of corridor densities, indicating that the proposed lightweight detector and HMTC strategy are feasible for deployment on embedded farm-side hardware.

## 4. Discussion

The proposed YOLO-pig model integrates three targeted modifications, namely, C3k2_RepViT, DySample, and Shape-IoU, into the YOLO11s baseline to address the specific challenges of pig detection in dense passage scenarios. Specifically, the C3k2_RepViT module prioritizes computational efficiency, yielding a 12.3% reduction in parameters and a 9.4% decrease in GFLOPs with minimal impact on accuracy. Meanwhile, DySample and Shape-IoU focus on enhancing detection fidelity through content-aware upsampling and geometry-sensitive bounding box regression, respectively. Their individual contributions to mAP_50-95_ (0.825 and 0.813 vs. the 0.791 baseline) suggest that spatial feature representation and regression precision represent orthogonal yet equally vital dimensions for performance improvement in this domain. The integrated YOLO-pig model achieves a peak mAP_50_ of 0.982 while maintaining a more compact footprint (8.28M parameters) than the baseline (9.41M), demonstrating that architectural efficiency and detection robustness are not mutually exclusive when modifications are selected to address scenario-specific constraints. This balance is particularly critical for precision livestock management applications, where edge-deployed systems must sustain high detection accuracy under the computational and memory limitations of farm-side hardware. Efficient use of limited computing resources is a recurring concern in real-time intelligent systems more broadly, where resource-allocation and efficiency-oriented frameworks have been explored to meet such constraints [31].

**Figure 11:**
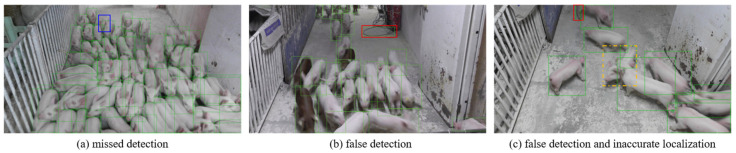
Representative failure cases of the detector. (**a**) Missed detection (blue box); (**b**) false detection on a background region (red box); (**c**) inaccurate localization (orange box).

Despite this favorable accuracy–efficiency balance, the detector still exhibits characteristic failure modes under the most challenging conditions, as illustrated in Figure 11. Missed detections (Figure 11a) occur mainly when pigs are heavily occluded by neighbors or truncated at the frame boundary; false detections (Figure 11b) are occasionally triggered by background structures that resemble pigs; and inaccurate localization (Figure 11c) arises when overlapping bodies make the precise extent of an individual ambiguous. These errors are infrequent and concentrated in the densest scenes, which is consistent with the residual gap in mAP@50-95. Importantly, because the downstream HMTC strategy counts on the basis of multi-frame temporal confirmation and trajectory verification rather than relying on any single-frame detection, such sporadic errors have limited impact on the final counting accuracy.

For the counting task, existing literature predominantly treats pig counting as a static detection task, focusing on frame-level density estimation within controlled, enclosed pens [32,33,34]. While these methods are effective in snapshot scenarios, they fail to capture the continuous flow of animals through passages, making them unsuitable for the transfer corridor applications addressed in this study. In contrast, some research has extended counting to the video domain via object tracking [16,17,18], achieving promising results under largely unidirectional flow, with reported accuracies of 99.44% [16] and 96.58% [17]. However, challenges remain for bidirectional counting, particularly in robustly registering reverse movements. The proposed HMTC strategy bridges this gap by achieving an exceptional overall accuracy of 99.56% and maintaining absolute precision (100.00% accuracy) in backward counting across all nine test sequences.

**Figure 12:**
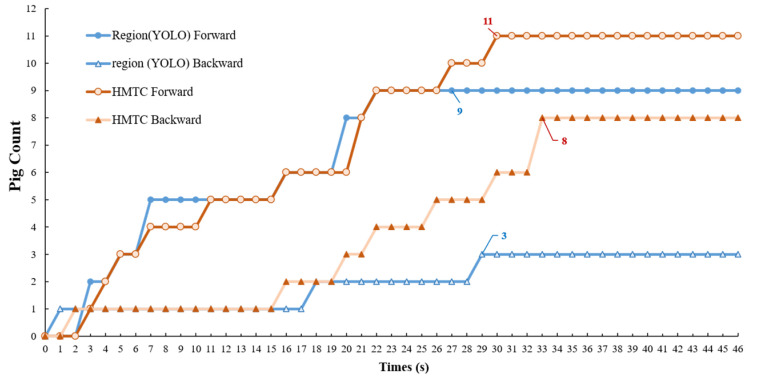
Cumulative pig counts of Region (YOLO) and HMTC for Video 4.

To investigate the mechanisms underlying the backward counting discrepancies, we conducted a failure case analysis of Videos 3, 4, and 8, which exhibited the lowest backward accuracy among all test sequences. As illustrated in Figure 12, the YOLO-based region method exhibits erratic forward count increments in Video 4 between approximately 6 and 20 s. This instability arises when pigs linger near the detection boundary, where repeated triggering leads to an overcount. In contrast, HMTC maintains a stable and monotonically increasing count throughout this interval, as the hysteresis band and multi-frame confirmation mechanism collectively suppress boundary oscillations.

**Figure 13:**
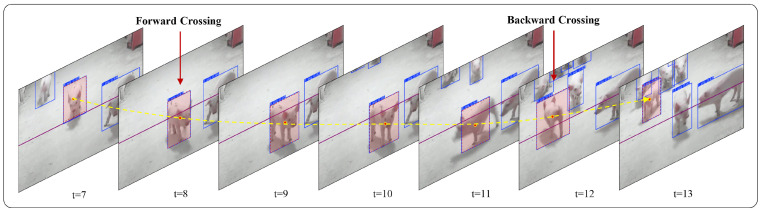
Sequential snapshots of failed backward counting in Video 3.

Moreover, a distinct failure mode was identified in Video 3, as shown in Figure 13, where an individual pig successfully crosses the detection zone in the forward direction but subsequently reverses its course within the same tracking session. Under the YOLO counting solution, this reverse crossing remains unregistered because the deduplication mechanism designed to prevent redundant forward counts also inadvertently blocks legitimate backward movements by the same animal. This logic introduces systematic undercounting of reverse movements that is difficult to mitigate in practice. A similar pattern is observed in Video 8 despite a substantially higher frequency of reverse movements. HMTC resolves this limitation by evaluating complete trajectory state transitions instead of relying on isolated boundary crossings, thereby ensuring consistent bidirectional registration regardless of track ID continuity.

While the proposed system demonstrated promising performance, several limitations should be acknowledged. Accordingly, the present results should be interpreted as a preliminary validation under a single transfer-corridor condition rather than as evidence of general applicability. The dataset was collected from a single facility focusing on weaned Landrace piglets, and the generalizability to other breeds, growth stages, or diverse farm layouts remains to be verified. Detection and tracking in other species can present distinct difficulties, such as the appearance-based identification challenges reported for dark-coated cattle [35]. In addition, although the complete pipeline was validated on an embedded edge device (NVIDIA Jetson Orin NX), where it sustained real-time operation without loss of counting accuracy, this evaluation was conducted on a single device type using offline video, so full on-farm integration with live camera streams remains to be assessed. Furthermore, the HMTC strategy remains dependent on the continuity of the underlying ByteTrack tracker, and its robustness under extremely high-density occlusion scenarios warrants further investigation. Future work should prioritize dataset diversification across broader husbandry environments, breeds, and growth stages, end-to-end deployment with live camera streams under real farm conditions, and the exploration of more advanced occlusion-handling techniques, so as to further strengthen the practical applicability of the proposed system.

## 5. Conclusions

In this study, we presented an integrated system for automated pig passage counting, combining an improved YOLO-based detection model with the proposed HMTC counting strategy. We incorporated three targeted modifications, namely, C3k2_RepViT, DySample, and Shape-IoU, into the YOLO11s baseline, yielding a compact detection model with 8.28M parameters that achieves a mAP_50_ of 0.982, demonstrating that detection accuracy and computational efficiency can be simultaneously improved through scenario-specific architectural design. The proposed HMTC strategy addresses the bidirectional counting challenge inherent to pig transfer corridor scenarios by integrating hysteresis-based region classification, multi-frame temporal confirmation, and trajectory-based verification, achieving an overall counting accuracy of 99.21% with no backward miscounts on the current test set, substantially outperforming the line- and region-based counting solutions built on the same detection and tracking backbone.

The results demonstrate that reliable automated pig passage counting is achievable under the tested transfer-corridor condition, including dense crowding, boundary lingering, and reverse movements. The complete pipeline was further validated on an embedded edge device (NVIDIA Jetson Orin NX), sustaining real-time operation (over 30 FPS) with counts identical to those obtained on the workstation, confirming that the system can be deployed on farm-side hardware without loss of counting accuracy. The proposed system provides a practical foundation for precision livestock management, supporting accurate animal inventory tracking during transfer operations. Future work will focus on extending the dataset to more diverse farm environments and integrating the system with broader livestock monitoring pipelines to further enhance its applicability in commercial production settings.

## 6. Patents

A Chinese invention patent application related to this work has been filed with the China National Intellectual Property Administration (CNIPA), titled “A bidirectional counting method, system, device, medium, and product for pig transfer passages” (Application No. 202610752230.6).

## Figures and Tables

**Table 1:** Summary of the pig passage video dataset.

No.	Duration (s)	Number of Images	Total Crossings	Forward Crossings	Backward Crossings
1	18	282	9	7	2
2	20	355	17	15	2
3	30	485	14	13	1
4	46	750	19	11	8
5	33	542	17	14	3
6	49	806	20	9	11
7	99	1594	76	64	12
8	37	637	44	22	22
9	12	201	38	38	0

**Table 2:** Parameter settings of the HMTC counting strategy.

Parameter	Value	Selection Basis
Base tracker	ByteTrack	Motion-based association robust to short occlusions, without appearance features; suited to real-time use
Upper/lower region thresholds	h/3 ± a/2	Centered on the detection line; defined relative to frame size for resolution independence
Region band height	h/15	Sets the width of the transition zone around the detection line
Hysteresis width	a/5	A fraction of the region band, set empirically to suppress boundary jitter around the detection line
Confirmation frames	3	A region change must persist for ≥2 frames to be accepted, rejecting single-frame noise
Minimum/maximum velocity	2.0/150.0 px·frame	Lower bound filters static jitter; upper bound rejects physically implausible jumps
Position-history length	10 frames	Window used for trajectory-based direction inference
Velocity-history length	5 frames	Smooths instantaneous velocity for direction voting
Maximum mid-zone dwelling	3s	Triggers trajectory-based re-inference for pigs lingering in the transition zone
Maximum consecutive anomalies	5	Number of anomalous frames tolerated before a track’s state is reset
Maximum absence frames	30 frames	A track is removed after disappearing for this many frames

**Table 3:** Comparison of IoU loss functions on the pig detection dataset.

IoU	P	R	mAP_50_	mAP_50-95_
CIoU	0.960	0.939	0.978	0.791
DIoU	0.943	0.942	0.976	0.783
GIoU	0.949	0.945	0.975	0.790
Shape IoU	0.961	0.932	0.975	0.813

**Table 4:** Ablation study of the proposed modules.

No.	C3k2_RepViT	DySample	P	R	mAP_50_	mAP_50-95_	Params(M)	GFLOPs
1			0.961	0.932	0.975	0.813	9.41	21.3
2	✓		0.954	0.940	0.976	0.800	8.25	19.3
3		✓	0.968	0.939	0.977	0.825	9.45	21.6
4	✓	✓	0.970	0.943	0.982	0.820	8.28	19.4

**Table 5:** Comparative counting performance of three methods across nine test videos.

Methods	TP (F/B)	FP (F/B)	FN (F/B)	AE (F/B)	ΣAE	ACC
Line (YOLO)	184/42	3/0	9/0	12/19	31	87.80%
Region (YOLO)	181/44	4/0	5/17	9/17	26	89.76%
HMTC	191/61	0/0	2/0	2/0	2	99.21%

**Table 6:** End-to-end latency and resource usage of the complete pipeline.

Resolution	Latency (ms)	FPS	RAM (GB)	GPU Shared (MB)
1080p	32.87	30.4	0.9	209
4k	79.49	12.6	1.2	292

## Data Availability

The data presented in this study are available on request from the corresponding author due to privacy restrictions of the farm facility.

## Abbreviations

The following abbreviations are used in this manuscript:

SSIM	Structural Similarity Index Measure
P	Precision
R	Recall
FPS	Frames Per Second
AE	Absolute Error
ACC	Accuracy
mAP	mean Average Precision
IoU	Intersection over Union
HMTC	Hysteresis-based Multi-frame Temporal Confirmation Counting Strategy
YOLO	You Only Look Once

## Appendix A

As shown in Table A1, YOLO11n achieves the lowest computational cost (2.6M parameters, 6.5 GFLOPs) but yields the weakest detection performance, with a mAP_50_ of 0.941 and mAP_50-95_ of 0.667, indicating insufficient capacity for dense pig detection scenarios. YOLO11m and YOLO11l deliver the strongest overall accuracy, with mAP_50-95_ reaching 0.827 and 0.838, respectively, but at substantially higher computational costs (20.1M/68.0 GFLOPs and 25.3M/86.9 GFLOPs) that are prohibitive for real-time edge deployment. YOLO11s strikes a favorable balance, achieving a competitive mAP_50_ of 0.978 and mAP_50-95_ of 0.791 with only 9.4M parameters and 21.5 GFLOPs, making it the most suitable baseline for subsequent improvements targeting both detection accuracy and deployment efficiency.

**Table A1:** Comparative results of YOLO series model in pig detection.

YOLO11	P	R	mAP_50_	mAP_50-95_	Params(M)	GFLOPs
n	0.933	0.879	0.941	0.667	2.6	6.5
s	0.960	0.939	0.978	0.791	9.4	21.5
m	0.965	0.935	0.979	0.827	20.1	68.0
l	0.959	0.947	0.979	0.838	25.3	86.9

As for edge deployment, the end-to-end latency is decomposed into six stages. Decode reads and decompresses each video frame; preprocess resizes and letterboxes the frame to the 640 × 640 network input and normalizes it; inference is the forward pass of the TensorRT detection engine; postprocess applies confidence filtering and NMS to the raw predictions; track association and result transfer (ByteTrack) matches detections to existing trajectories, assigns identities, and moves the results from GPU to CPU; and HMTC performs the hysteresis-based region classification, multi-frame confirmation, and trajectory verification that produce the directional counts.

**Table A2:** Per-stage latency and resource usage of the complete pipeline on the Jetson Orin NX (TensorRT FP16, 640 × 640 inference, software decoding), on the densest sequence (video7) at 1080p and native 4K. Each value is the mean of three runs after a 30-frame warm-up; memory is the increment over idle (jtop).

Stage (ms/frame)	1080p	4k
Decode	8.60	27.98
Preprocess	4.82	14.15
Inference	7.48	7.40
Postprocess	4.76	4.80
Track assoc. & transfer	7.05	25.01
HMTC	0.16	0.16
End-to-end latency	32.87	79.49
End-to-end FPS	30.4	12.6
Incremental RAM (unified)	0.9 GB	1.2 GB
Incremental GPU shared	209 MB	292 MB
